# The correlates of post-surgical haematoma in older adults with proximal femoral fractures

**DOI:** 10.1007/s40520-023-02354-6

**Published:** 2023-02-11

**Authors:** Carmelinda Ruggiero, Giulio Pioli, Rosario Petruccelli, Marta Baroni, Raffaella Prampolini, Paolo Pignedoli, Pierluigi Antinolfi, Giuseppe Rinonapoli, Michele Cappa, Virginia Boccardi, Chiara Bendini, Patrizia Mecocci, Auro Caraffa, Ettore Sabetta

**Affiliations:** 1 Orthogeriatric and Geriatric Unit, Department of Medicine and Surgery, Gerontology and Geriatric Section, S. Maria Misericordia Hospital, University of Perugia, S. Andrea delle Fratte, 06156 Perugia, Italy; 2 Orthogeriatric and Geriatric Unit, Department of Neuromotor Physiology and Rehabilitation, ASMN-IRCCS Hospital, Viale Risorgimento 80, 42123 Reggio Emilia, Italy; 3 Orthopedic and Trauma Unit, Department of Medicine and Surgery, Orthopedic and Trauma Unit, Department of Medicine and Surgery, 06156 Perugia, Italy; 4 Orthopaedic Unit, Department of Neuromotor Physiology and Rehabilitation, ASMN-IRCCS Hospital, Viale Risorgimento 80, 42123 Reggio Emilia, Italy

**Keywords:** Haematoma, Proximal femoral fractures, PPIs, SSRI, Ultrasound detection

## Abstract

**Background:**

Little is known about the incidence of haematoma, and clinical correlates among orthogeriatric patients.

**Aims:**

This study aims to describe the incidence of haematoma after surgical repair of hip fracture and to identify the clinical correlates of haematoma among orthogeriatric patients.

**Methods:**

Two orthopaedic surgeons and a dedicated operator using ultrasound technique, each other in blindness, evaluated 154 orthogeriatric patients during their hospital stay. All patients received a comprehensive geriatric assessment. We investigated the concordance between clinical diagnosis and ultrasound detection of haematoma, and then we explored the clinical correlates of the onset of post-surgical haematoma.

**Results:**

Blood effusion at the surgical site was detected in 77 (50%) patients using ultrasound technique; orthopaedic surgeons reached a clinical agreement about post-surgical haematoma in 18 (23%) patients. The sensitivity of clinical evaluation was 0.66, and the specificity was 0.70. Independent of age, clinical, pharmacological, and surgical confounders, proton pump inhibitors (PPIs) were associated with post-surgical haematoma (OR 2.28; 95% CI 1.15–4.49). A tendency towards association was observed between selective serotonin reuptake inhibitors and post-surgical haematoma (OR 2.10; 95% CI 0.97–4.54),

**Conclusions:**

Half of older patients undergoing surgical repair of proximal femoral fracture develop a post-surgical haematoma. Clinical assessment, even if made by senior orthopaedic surgeons, underestimates the actual occurrence of post-surgical haematoma compared to ultrasound detection. Ultrasound technique may help to detect haematoma larger than 15 mm better than clinical assessment. PPIs’s use is a risk factor for post-surgical haematoma independent of several medical and surgical confounders.

## Background

Hip fracture is a common orthopaedic disease, with an increasing demand for surgery in older patients and an incidence expected to increase in the following decades [1]. Hip fracture harms older adults and their relatives’ quality of life due to catastrophic loss of independence, morbidity, permanent disability following the event, and death. Hip fracture causes the most significant cost burden on healthcare services worldwide [2, 3].

Older patients undergoing surgery due to hip fracture are more likely to suffer multimorbidity, frailty, cognitive and functional impairment, and experience a high risk of complications [4]. The orthogeriatric comanagement improves the patients’ frailty and fragility fracture care and, per standard, focuses on anticoagulation, antiplatelets, and other factors contributing to perioperative bleeding, haemorrhage, and post-surgical haematoma [5].

In today’s clinical practice, the initial fracture haematoma with the local inflammatory response is the beginning of the healing process. Whenever possible, the original haematoma formed upon injury should be conserved during fracture treatment to benefit from the inherent healing potential [6].

Post-surgical haematoma is a different condition that forms after the original fracture haematoma is removed. Post-surgical haematoma is a visible subcutaneous blood and cloth collection exceeding the wound’s limits. It is a common complication of major surgery, usually associated with inadequate haemostasis [7]. The length of the incision and the skin blood flow are surgical parameters that could influence the severity of soft tissue damage and the risk of haematoma [8]. After hip fracture surgery, the haematoma is usually small, self-limiting, and self-resolving. Clinically evident haematoma presents oedema, ecchymosis, and serosanguinous wound drainage. It may cause pain and stiffness, impair mobility recovery, or lead to drainage. In the worst scenario, this postoperative complication may increase the risk of surgical site infection [9], requiring antibiotics and a prolonged hospital stay. Ultrasonography is helpful in the detection of post-surgical haematoma [10]. However, haematoma characteristics are variable, depending on the time, amount of blood loss, and the transducer frequency.

To date, the orthopaedic surgeon usually diagnoses post-surgical haematoma based on the clinical evaluation of the surgical site. There are currently no data demonstrating if surgical parameters, including the length of the incision and the skin blood flow, could influence the severity of soft tissue damage with the risk of haematoma. Some studies focussed on haematoma post-hip fracture surgery. They confirmed the role of single main risk factors for haematoma, such as antiplatelets and anticoagulants [11, 12]. Besides single drugs, few studies investigated the role of comorbidity and polypharmacy in post-surgical haematoma development and persistence in patients undergoing hip fracture repair. The study examines the relationship between patients’ clinical and surgical characteristics and postoperative haematoma among orthogeriatric patients co-managed by orthopaedic surgeons and geriatricians. Then, we evaluate the incidence of haematoma after hip fracture surgery by comparing the detection based on clinical signs with those based on ultrasound (US) technique.

## Materials and methods

### Study design and sample

We conducted a prospective cohort study in an orthogeriatric comanagement acute care ward. Participants were all consecutive patients admitted to the acute care ward due to proximal femoral fragility fracture aged 65 years or older from June 2016 to May 2017. The only exclusion criteria were patients who did not receive surgical repair of fracture or those with short life expectancy due to terminal illness. The study and all procedures were conducted in accordance with the ethical standards of the responsible committee on human rigths and with the Helsinki Declaration of 1975, revised in 2000 and 2008 and approval was obtained from the ethics committee of the regional healthcare system with registration number 1465 (EMAFEM_GERI15_22).

### Data collection

*Baseline characteristics.* The information recorded on admission included: age, gender, living arrangements (home, institution), type of fracture (extra-capsular or intra-capsular), functional and cognitive status, comorbidity, and severity of illness. Pre-fracture functional status (2 weeks before) was measured using the 6-item activities of daily living (ADL) and the 8-item instrumental activities of daily living (IADL) [13, 14]. Each item was logged as zero in case of total or partial assistance and as one in case of complete independence. Cognitive status was assessed by the Short Portable Mental Status Questionnaire (SPMSQ, range 0–10) [15]. Patients with an adjusted SPMSQ score of three or more errors were classified as having cognitive impairment. Medical burden and comorbidity were measured using the Charlson index [16]. Nutritional status was assessed through Mini Nutritional Assessment short-form (MNA-SF) [17].

Moreover, as a marker of sarcopenia, we measured handgrip strength which can be assessed in bedridden patients and is considered an index of whole-body resilience to ageing. Handgrip strength was measured using a JAMAR hand dynamometer (Model BK-7498, Fred Sammons Inc, Brookfield, Ill). Patients were in the supine position and encouraged to exhibit the greatest possible force; the best value of 3 assessments of the dominant hand was used for the analysis.

Drugs. All drugs taken by patients were recorded, particularly anticoagulation and antiplatelet therapy, and all medications are known to affect platelet aggregation, such as nonsteroidal anti-inflammatory drug, or able to inhibit CYP2C19 enzyme such as proton pump inhibitor (PPIs), Selective Serotonin Reuptake Inhibitors (SSRI), fluconazole, voriconazole, ciprofloxacin, carbamazepine, oxcarbazepine. Moreover, data were collected on preoperative and postoperative LMWH therapy for venous thromboembolism prophylaxis.

Surgery. Data related to surgery were gathered, including surgical delays (time from admission to surgery), type of surgery (particularly prosthesis or intramedullary nail), type of anaesthesia, duration of surgery (h), and placement of surgical drainage.

### Assessment of haematoma

A dedicated radiologist assessed patients’ surgical sites for the detection of post-surgical haematoma using the US technique. The radiologist’s evaluated the surgical site for haematoma at the bedside based on a semi-quantitative estimation of the volume on the third postoperative day and repeated it on the fifth postoperative day. The surgical site was examined using a LOGIQ S8 US system and a ML6-15 probe (GE Healthcare, Chalfont St Giles, United Kingdom). The same operator carried out the US evaluation of the hip surgical site, scanning hip anteriorly and laterally both in longitudinal and transverse planes, emphasising the region of surgical scar. The haematoma was defined as an echogenic area within the surgical area, and the maximum depth of the thickness was recorded in millimetres (mm); we did not record haematoma’s length because it is not more relevant than a collection limited to a few cm cephalo-caudal. The clinical evaluation was made blind by two independent senior orthopaedic surgeons. Typical signs were fullness and pain, bluish or purple discoloration of the underlying skin, and bleeding. The clinical agreement on the absence of haematoma, disagreement about haematoma’s presence, and agreement on haematoma’s presence was defined after two senior surgeons’ independent assessment.

### Statistical analysis

The study sample size was defined according to a priori power analysis based on a population size of 350 patients, those treated because of a hip fracture over 1 year at our site, with 30% as proportion of the event. Assuming, therefore, that 30% of the subjects in the population have the factor of interest, the study would require a sample size of 140 for estimating the expected proportion with 10% absolute precision and 99% confidence limits. In the enrolment, we considered also a 10% drop-out rate. The type of distribution of the variables was checked before analysis by Shapiro–Wilk test. Categorical variables were expressed in percentages, and continuous variables were reported as mean ± standard deviation given.

One-way analysis of variance, Pearson’s χ^2^ test, and the Mann–Whitney *U* test were used to examine differences in patients’ baseline characteristics or crude data between patients with and without haematoma. A multivariate logistic regression model was designed and executed to identify risk factors for developing a haematoma. Odds ratios (OR) and 95% confidence intervals (CIs) were calculated. Statistical significance was set at *p* < 0.05. Statistical analysis was performed with SPSS 22.0 for Windows (SPSS Inc., Chicago, IL). Moreover, the sensitivity and specificity of haematoma’s clinical detection were calculated compared to US diagnosis as a gold standard. Cohen’s kappa coefficient was used to measure the inter-rater agreement of the assessment by two orthopaedic surgeons.

## Results

### Detection of post-surgical haematoma based on clinical examination and US technique

Data from 154 subjects are available and presented in Table 1. The majority of patients experienced displaced femoral neck (38.4%) and pertrochanteric femoral fractures (45.4%), and more than half of them underwent hip fracture fixation (53%). Blood effusion at the surgical site was detected in 77 (50%) patients using the US technique within the 3rd postoperative day. The thickness of haematoma was 10 mm and more in 77% of the cases, 15 mm and more in 56%, and 20 mm and more in 49% of patients. The distribution of post-surgical haematoma was similar across the four classes of proximal femoral fractures (Table 1). In the overall sample, we found a correlation between the US evidence of post-surgical haematoma and clinical disagreement (45%) between the orthopaedic surgeons on the presence of a haematoma. On the contrary, the surgeons’ clinical agreement about the absence of haematoma was reached in 60% of cases when the haematoma was undetected by the US (Table 2). The correlation between surgeons’ agreement about the absence of the haematoma and undetected haematoma using the US was independent of the size. The highest surgeons’ agreement about the presence of haematoma (32%) was achieved when the size of haematoma was > 20 mm by the US, without an actual improvement among the different sizes of haematoma (Table 2). Overall, orthopaedic surgeons’ clinical detection of blood effusion was lower than that provided by the US technique (23% vs. 50%, p 0.002). When two senior orthopaedic surgeons agreed, sensitivity was 0.66 and specificity 0.70. Moreover, clinical judgement agreement between the two surgeons remained very low (64%, *K* value 0.190, *p* = 0.006). The results did not improve when the detection of blood effusion with a thickness of more than 15 or 20 mm was considered.

**Table 1:** The distribution of post-surgical haematoma in the entire sample and according with the classification of femoral fractures

Type of femoral fracture, *N* (%)	Haematoma (all) *N* (%)	Haematoma (thickness > 10 mm) *N* (%)	*p* value
Undisplaced femoral neck fractures, 12 (7.8)	7 (58%)	6 (50%)	0.389
Displaced femoral neck fractures, 59 (38.4)	29 (49%)	25 (42%)	0.304
Pertrochanteric fractures, 70 (45.4)	35 (50%)	25 (36%)	0.143
Subtrochanteric fractures, 13(8.4)	6 (46%)	6 (46%)	0.500

**Table 2:** The relationship between clinical assessment of haematoma by two orthopaedic surgeons and ultrasound detection of haematoma in the entire sample and according with thickness of the haematoma

	Entire sample *N* (%)	Clinical agreement on the absence of haematoma *N* (%)	Clinical disagreement on the presence of haematoma *N* (%)	Clinical agreement on the presence of haematoma *N* (%)
Haematoma detected by ultrasound (all)	77 (50%)	24 (31%)	35 (45%)	18 (23%)
Haematoma not detected by ultrasound (all)	77 (50%)	46 (60%)	25 (32%)	6 (8%)
Haematoma > 10 mm detected by ultrasound	57 (37%)	14 (25%)	28 (49%)	15 (26%)
Haematoma > 10 mm not detected by ultrasound	97 (63%)	56 (58%)	32 (33%)	9 (9%)
Haematoma > 15 mm detected by ultrasound	43 (28%)	10 (23%)	21 (49%)	12 (28%)
Haematoma > 15 mm not detected by ultrasound	111 (72%)	60 (54%)	39 (35%)	12 (11%)
Haematoma > 20 mm detected by ultrasound	28 (18%)	7 (25%)	12 (43%)	9 (32%)
Haematoma > 20 mm not detected by ultrasound	126 (82%)	63 (50%)	48 (38%)	15 (12%)

### Correlates of a post-surgical haematoma

Table 3 reports the characteristics of patients with femoral fractures according to the absence or post-surgical haematoma presence. The groups were similar regarding age, gender, comorbidity, polypharmacy, nutritional status, functional status, fracture type, surgical repair strategy, and anesthesiologist approach. Variables related to surgical repairing, such as delay to surgery, type of surgery, surgery duration, type of anaesthesia, and drains’ insertion, were also similar between groups. Warfarin anticoagulation before surgery and antiplatelet drugs, irrespective of the type of drug (only five patients were on clopidogrel and four on ticlopidine), were equally distributed. Most patients received LMWH for thromboprophylaxis (only three patients received fondaparinux) starting before surgery if the intervention was scheduled the day after admission or later or after surgery if the patient received intervention the day of admission. The haematoma rate was not significantly different between patients receiving or not receiving pharmacologic prophylaxis before surgery.

**Table 3:** The characteristics of the participants grouped according with detection of post-surgical haematoma

	Overall sample (n: 154)	Absence of haematoma (n: 77)	Presence of haematoma (n: 77)	*p* value*
Comprehensive assessment				
Age, year (mean ± SD)	85.1 ± 7.1	84.2 ± 7.0	86.0 ± 7.1	0.109
Gender, men (%)	20.4	19.5	22.1	0.692
Living in institution, (%)	5.8	7.8	3.9	0.304
Charlson index, score (mean ± SD)	1.6 ± 1.6	1.3 ± 1.4	1.8 ± 1.9	0.139
Drugs (mean ± SD)	5.5 ± 2.9	5.6 ± 3.2	5.5 ± 2.8	0.851
MNA-SF, score (mean ± SD)	10.6 ± 3.0	10.7 ± 3.2	10.6 ± 2.9	0.902
ADL, score (mean ± SD)	4.5 ± 1.7	4.5 ± 1.8	4.5 ± 1.6	0.963
IADL, score (mean ± SD)	4.2 ± 3.1	4.1 ± 3.2	4.3 ± 3.1	0.603
Hand grip, kg (mean ± SD)	14.5 ± 6.8	14.8 ± 6.8	14.3 ± 6.9	0.643
SPMSQ score, (mean ± SD)	6.4 ± 3.4	6.4 ± 3.4	6.5 ± 3.4	0.812
Surgical characteristics				
Surgery < 24 h (%)	49	51	48	0.687
Surgery 24–48 h (%)	42	48	52	0.845
Surgical delay, hour, (mean ± SD)	34 ± 19	34 ± 22	33 ± 15	0.560
Extra capsular fracture (%)	54	54	53	0.872
Type of surgery, prosthesis (%)	46	45	49	0.936
Length of surgery, hours (mean ± SD)	1.0 ± 0.3	1.1 ± 0.3	1.0 ± 0.3	0.462
Anaesthesia, general (%)	11	14	9	0.302
Drainage, (%)	55	55	56	0.832
Factors related with the management of the acute phase				
Anticoagulation, (%)	16	19	15	0.562
Antiplatelet, (%)	41	37	48	0.208
Selective serotonin reuptake inhibitors, (%)	27	19	34	0.046
Proton pump inhibitors, (%)	43	32	55	0.006
Preoperative LMWH, lapse of hours to intervention (mean ± SD)	16.5 ± 2.4	16.1 ± 2.3	17.3 ± 2.4	0.739
Preoperative LMWH not administered (%)	13	13	8	
Postoperative LMWH, lapse of hours from intervention (mean ± SD)	7.5 ± 2.4	7.5 ± 1.5	7.6 ± 3.2	0.767
Length of hospital stay, day (mean ± SD)	10.5 ± 3.5	10.2 ± 3.6	10.8 ± 3.4	0.354
Blood transfusion, unit (mean ± SD)	1.5 ± 1.3	1.3 ± 1.3	1.6 ± 1.3	0.188
Delirium, incident event (%)	43	38	49	0.145

*ADL* activities of daily living, *IADL* instrumental activities of daily living, *SPMSQ* Short Portable Mental Status Questionnaire, *MNA-SF* Mini Nutritional Assessment short-form

*p value are from differences between groups

The distribution of participants with detectable US haematoma was similar across the fractures’ type and the lapses of time from the end of the surgery and the beginning of LMWH thromboprophylaxis. Even though the guideline suggests starting LMWH thromboprophylaxis 6 h after surgery, in practice, all patients received post-surgery thromboprophylaxis after a variable lapse of time from the intervention, mainly depending on the accomplishment with the surgical list. Patients receiving LMWH thromboprophylaxis after 8 h from the end of the surgery only tended to experience a slight reduction in the incidence of post-surgical haematoma (*p* 0.625). A detectable US haematoma was found in 52% of participants receiving post-surgery thromboprophylaxis within 6 h or 6–8 h from the end of the surgery, respectively. Participants receiving thromboprophylaxis within 8–10 h and after 10 h from the end of surgery experienced haematoma in 48% and 46% of cases, respectively.

Among drugs affecting the CYP2C19 enzyme, we included PPIs and SSRI in the analyses since other medications listed in the method section were not taken at all or taken in by very few patients. However, these two drugs were the main significant risk factors for haematoma among the recorded variables. In the multivariate analysis, independent of age, sex, type of fracture, surgical delay, and drugs, including anticoagulation, antiplatelets, SSRI antidepressant, we found that PPIs increased 2.5 times the risk of post-surgical haematoma (OR 2.28, 95% CI 1.15 ± 4.49, *p* = 0.018) and SSRI about 2.1 times closed to significant value (OR 2.10, 95% CI (0.97 ± 4.54, *p* = 0.059) (Table 4). No patient underwent surgical evacuation of the haematoma. Length of stay, and overall blood transfusions, were not significantly affected by the presence of a post-surgical haematoma.

**Table 4:** The multivariate analysis of the association between patients’ characteristics and post-surgical haematoma

	OR (95% CI)	*p* value
Age	1.04 (0.99–1.10)	0.101
Male gender	1.23 (0.53–2.84)	0.625
Extracapsular fracture	0.90 (0.53–1.52)	0.686
Surgical delay > 24 h	1.08 (0.52–2.25)	0.842
Anticoagulation use	0.68 (0.26–1.80)	0.437
Antiplatelet use	1.29 (0.64–2-61)	0.483
SSRI use	2.10 (0.97–4.54)	0.059
PPI use	2.28 (1.15–4.49)	0.018

## Discussion

About half of older patients undertake surgery because proximal femoral fractures develop post-surgical haematoma at the surgical site. Even after adjudication by two senior orthopaedic surgeons, clinical detection of haematoma remains significantly lower than that detected by the US technique, with a high rate of clinical disagreement. Focussing on post-surgical haematoma seen by the US, PPIs’s use results in a statistically significant predictor of post-surgical haematoma independent of age, clinical, other pharmacological, and surgical confounders. A tendency towards statistically significant association appears between SSRI and post-surgical haematoma.

Post-surgical haematoma is empirically considered a risk factor for infection at the surgical site, causing patients morbidity and mortality despite adequate antibiotic treatment. Post-surgical haematoma may increase the hospital length of stay and the readmission rates and may negatively impact referral hospitals, raising healthcare costs [9, 18, 19].

The initial haematoma adjacent to damaged tissues is considered the best immunological environment for bone healing. In the surgical phase, the open reduction and internal fixation of closed fractures inevitably disrupt the initial haematoma, as would debridement and irrigation of open fractures. Then, the matured initial haematoma is replaced by a naïve post-surgical haematoma, whose composition is more like that of the peripheral blood. The immune cell population of post-surgical haematoma differs from that of primary haematoma [6, 20]. It does not fit into the surrounding tissues’ healing stage and may affect the progenitor cells. The mismatch between post-surgical haematoma and surrounding tissue may delay the fracture healing, may hinder the healing process and increase the risk of post-surgical infections [6].

Fracture reduction in human trauma patients is routine in clinical practice, and post-surgical haematoma prevention may offer opportunities to prevent wound leakage and prosthetic joint infection [21–23]. However, few studies investigated post-surgical haematoma incidence and risk factors in patients with proximal femoral fractures, mainly due to diagnostic difficulties and multifactorial genesis of bleeding. In this scenario, the orthogeriatric management may help by providing multisystemic characterisation of patients and offering interventions, including those affecting coagulation, based on the individual comprehensive assessment [5].

In our study, the clinical evaluation of haematoma has a low sensitivity and specificity with a high number of false positives and negatives, even if two senior orthopaedic surgeons adjudicated the event. A negative US exam correlates with the senior surgeons’ agreement for the absence of haematoma; instead, an US-detected haematoma is associated with a higher rate of disagreement with senior surgeons’ assessment, whatever the size. In studying factors related to a haematoma, we consider the US technique likely a strength so that we could carefully detect the presence and size of haematoma, thus overcoming the clinical disagreement between surgeons. In addition, the comprehensive geriatric assessment could be an additional study’s strength, offering the possibility to explore the role of non canonical factors potentially involved in the development of haematoma, despite biological plausibility [24].

Fagotti et al. [24] proposed a classification of haematoma based on the area (cm^2^) and skin incision length that showed higher intra- and inter-rater reliability. The correct identification of a haematoma could provide an outline for proper therapeutic measures and serve as an instrument for optimising communication between surgeons and healthcare providers. Many haematomas are small and reabsorb on their own. Still, some can become large enough to cause immediate consequences such as sciatic nerve palsy, excessive pain, swelling, and persistent wound drainage. Thus, we suggest a US evaluation to diagnose post-surgical haematoma, especially if requested by the clinical course. US let us confirm the absence of haematoma after surgery and avoid the failure of clinical diagnosis. However, US imaging at the surgical site can be complex and challenging after femoral surgery, even more given the need for consensus about the US criteria for clinical relevance. Moreover, to our knowledge, no unique data are available to define and grade the post-surgical haematoma.

Unlike previous studies, the prevalence of post-surgical haematoma was similar between extra-capsular and intra-capsular femoral fractures [25, 26]. A tendency for a higher incidence of post-surgical haematoma > 10 mm was found in the subtrochanteric fractures. The prevalence of post-surgical haematoma was similar among different surgical techniques, including arthroplasty and fixation, length of surgery, type of anaesthesia, and drainage. However, the relationship between post-surgical haematoma and early failure of hip fracture fixation needs to be investigated in the short and long term [27].

We acknowledge that patients included in our study did not receive mini-incision technique, which usually results in less bleeding or trauma to the femur’s soft tissues than the standard technique [8, 28]. Most orthopaedic surgeons use and leave drainages in place for 24 h, applying teachings from general surgery. On one side, it may reduce the incidence of surgical wound complications, including haematoma, slow internal bleeding, delayed healing, and wound dehiscence [29]. On the other side, no proof of an unfavourable effect on the risk of infection is available.

Our findings do not support the drainage placement to reduce the incidence of haematoma, nor the relationship between drainage placement and the incidence of infection, requiring more investigation among older frail patients [30].

Orthopaedic surgeons extensively prescribe PPIs as prophylaxis against potential life-threatening gastrointestinal complications such as stress ulcers during the treatment of fractures [31]. However, the relationship between PPIs use and haematoma risk is a new finding from our study, with evidence that PPIs increases the risk of post-surgical haematoma by 2.5 times. Although we acknowledge that this result needs to be confirmed on a larger sample, previous studies showed that patients taking PPIs have an increased the risk of non-union in tibial and femoral shaft fractures compared with non-users [32]. Another recent report has shown that patients on PPIs may have higher non-union rates following an anterior cervical discectomy and fusion [33].

From biological standpoint, PPIs seems interfering with the healing process affecting the osteoclastic activity during its early phases and the expression of osteocalcin and osteoprotegerin in animal models [34], leading to non-unions in cervical spine fusion procedures [35].

SSRIs are among the most commonly prescribed medications in the elderly for the treatment of depression. These agents are the preferred choice over other classes of antidepressants owing to their more favourable safety profile.

We confirm previous findings showing that SSRIs’ perioperative use is associated with a higher risk for adverse events and poorer post-surgical outcomes, including bleeding and the need for transfusions [36]. A literature review confirmed a correlation between ongoing SSRI therapy and intraoperative risk of bleeding. Six out of seven studies in orthopaedic surgery (e.g. five for hips, one for all orthopaedic procedures, and one for spinal fusion) report significantly higher rates of intraoperative blood loss or need for transfusions in SSRI users compared with non-users [37].

SSRIs selectively inhibit presynaptic serotonin (5-HT) reuptake, and it is supposed that they increase the risk of abnormal bleeding events by inhibiting the uptake of serotonin into platelets leading to impairment in the platelet haemostatic response. Currently, it appears that serotoninergic mechanisms play an essential role in haemostasis [38]. As in previous studies, we confirm that anticoagulation therapy and antiplatelet drugs do not increase the risk of blood effusion after surgery [39, 40]. In our research, LMWH thromboprophylaxis is not an independent risk factor for post-surgical haematoma.

On the contrary, previous findings showed an increased risk of wound haematoma without differences concerning mortality, thromboembolic phenomena, haemorrhagic complications, or wound complications [41]. We acknowledge that the orthogeriatric comanagement of patients makes our study different from previous ones. We assume that the orthogeriatric comanagement may reduce the risk of bleeding complications by addressing and managing the factors related to the intrinsic frailty of this subgroup of surgical patients [42, 43]. However, a larger sample with a higher statistical power could better clarify the significance of the small differences in the rate of haematoma found between patients receiving LMWH before and after 8 h from the end of surgery. Although adjustments for fracture type, surgical delay, preoperative use of antiplatelet, and SSRI drugs, other factors related with the blood vessel fragility, connective tissue weakness, or the surgical procedures, not explored in this study, may probably account for the risk of haematoma.

Ultimately, as previously shown by our group, tranexamic acid might reduce post-surgery blood loss. Still, we did not investigate the relationship with the formation or prevention of post-surgical haematoma [44]. In this study, we cannot add to this point since no patient received tranexamic acid at the time of surgery. Post-surgical haematoma did not require specific treatment, and we only monitored its evolution and predictors in the short-term period. Further studies are needed to investigate the relationship between haematoma and surgical or clinical complications in the mild and long term from surgery.

To the best of our knowledge, this is the first clinical study analysing the incidence and the predictors of post-surgical haematoma among older hip fracture adults that have been managed according to the gold standard model of care. The detailed clinical and surgical descriptions of patients reduced the risk of confounding bias, then our regression analyses were also adjusted to several factors such as to ASA score, type of fractures, timing of surgery, comorbidity, polypharmacy, and length of hospital stay. We acknowledge that the findings need to be replicated by further studies, and the prognosis of post-surgical haematoma is still unknown. The observational design of this study cannot assure a causal effect relation between PPIs use and the risk of post-surgical haematoma. In addition, the sample size was relatively small, and the regression analysis did not include other variables that could be related to post-surgical haematomas such as the level of physical activity. Ultimately, the patient’s prognosis of haematoma is unknown and further investigations are needed to understand how to improve the management of this subgroup of older hospitalised patients.

## Conclusions

One out of two older adults develops a post-surgical haematoma after surgery because of a femoral fracture. The US technique may improve the diagnosis of post-surgical haematoma as compared to the clinical assessment, which even if made by two senior orthopaedic surgeons, underestimates its real occurrence. Independent of several medical and surgical factors, the chronic use of PPIs remains significantly associated with the risk of post-surgical haematoma. Conversely, we did not find a statistically significant association between anticoagulants, including LMWH, antiplatelet drugs and increased blood effusion. Given the increasing burden of proximal femoral fractures in older adults and the potential clinical implications of post-surgical haematoma in the healing process and functional recovery of older people receiving surgery, more studies in orthogeriatric settings are needed to confirm our findings.

## Data Availability

Datasets on which the conclusions of the manuscript rely are deposited in publicly available repositories. All materials and data are available on request.
