# Correction: The correlates of post-surgical haematoma in older adults with proximal femoral fractures

**DOI:** 10.1007/s40520-023-02626-1

**Published:** 2023-12-08

**Authors:** Carmelinda Ruggiero, Giulio Pioli, Rosario Petruccelli, Marta Baroni, Raffaella Prampolini, Paolo Pignedoli, Pierluigi Antinolfi, Giuseppe Rinonapoli, Michele Cappa, Virginia Boccardi, Chiara Bendini, Patrizia Mecocci, Auro Caraffa, Ettore Sabetta

**Affiliations:** 1 Orthogeriatric and Geriatric Unit, Department of Medicine and Surgery, Gerontology and Geriatric Section, S. Maria Misericordia Hospital, University of Perugia, S. Andrea delle Fratte, 06156 Perugia, Italy; 2 Orthogeriatric and Geriatric Unit, Department of Neuromotor Physiology and Rehabilitation, ASMN-IRCCS Hospital, Viale Risorgimento 80, 42123 Reggio Emilia, Italy; 3 Orthopedic and Trauma Unit, Department of Medicine and Surgery, Orthopedic and Trauma Unit, Department of Medicine and Surgery, 06156 Perugia, Italy; 4 Orthopaedic Unit, Department of Neuromotor Physiology and Rehabilitation, ASMN-IRCCS Hospital, Viale Risorgimento 80, 42123 Reggio Emilia, Italy

**Correction: Aging Clinical and Experimental Research (2023) 35:867–875** 10.1007/s40520-023-02354-6

In this article the author name Ettore Sabetta was incorrectly written as Ettore Sabbetta.

The original article has been corrected.

